# Nanometer Interlaced Displacement Metrology Using Diffractive Pancharatnam-Berry and Detour Phase Metasurfaces

**DOI:** 10.1021/acsphotonics.4c01451

**Published:** 2024-11-30

**Authors:** Nick Feldman, Kian M. M. Goeloe, Arie J. den Boef, Lyubov V. Amitonova, A. Femius Koenderink

**Affiliations:** † Department of Information in Matter and Center for Nanophotonics, AMOLF, Science Park 104, 1098 XG Amsterdam, The Netherlands; ‡ Advanced Research Center for Nanolithography (ARCNL), Science Park 106, 1098 XG Amsterdam, The Netherlands; § Department of Physics and Astronomy, and LaserLaB, Vrije Universiteit, 1081 HV Amsterdam, The Netherlands; ∥ ASML Netherlands B.V., De Run 6501, 5504 DR Veldhoven, The Netherlands

**Keywords:** metasurfaces, metrology, Fourier microscopy, polarimetry, Pancharatnam-Berry phase, detour phase

## Abstract

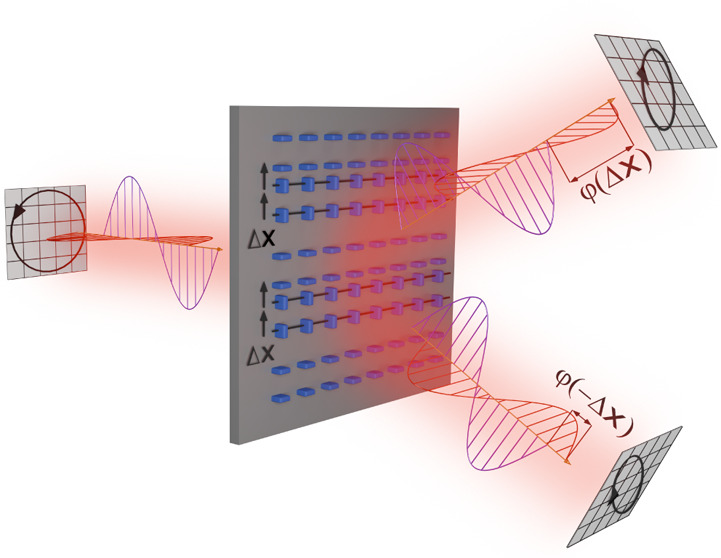

Resolving structural misalignments on the nanoscale is of utmost importance in areas such as semiconductor device manufacturing. Metaphotonics provides a powerful toolbox to efficiently transduce information on the nanoscale into measurable far-field observables. In this work, we propose and demonstrate a novel interlaced displacement sensing platform based on diffractive anisotropic metasurfaces combined with polarimetric Fourier microscopy capable of resolving a few nanometer displacements within a device layer. We show that the sensing mechanism relies on an interplay of Pancharatnam-Berry and detour phase shifts and argue how nanoscale displacements are transduced into specific polarization signatures in the diffraction orders. We discuss efficient measurement protocols suitable for high-speed metrology applications and lay out optimization strategies for maximal sensing responsivity. Finally, we show that the proposed platform is capable of resolving arbitrary two-dimensional displacements on a device.

## Introduction

The field of optical metrology^1,2^ covers a range of measurement techniques in which light is used as the main information carrier, finding applications in, for instance, superresolution imaging,^3−5^ gravitational wave detection,^6^ and medical diagnostics.^7^ In these technologies, a physical property of an object or specimen affects fundamental properties of an electromagnetic wave, such as polarization, amplitude, phase, or propagation direction, which is subsequently read out in a suitable measurement protocol. In optical metrology, the goal is to gather as much information as possible about this single unknown property of interest while assuming prior knowledge and control over all other relevant parameters. By virtue of this prior knowledge, the single unknown parameter can be deduced with deeply subwavelength resolution, which highlights a crucial difference between the fields of optical metrology and imaging, where in the latter, no such prior knowledge is assumed at the cost of a resolution bound defined by the diffraction limit. Optical metrology techniques to measure spatial positions, displacements, sizes and shapes on the nanoscale are therefore an indispensable tool in modern-day semiconductor device manufacturing processes.^8^ Nanoscale structures which are lithographically defined in a resist and subsequently transferred into a device wafer are routinely checked for potential fabrication mishaps such as defects, relative alignment errors between devices and device layers, surface roughness,^9,10^ size variations that might occur due to overexposure or overetching, and shape errors. To keep up with the exponentially decreasing trend in the feature size of these structures, known as Moore’s law,^11^ there is a stringent need for refined and highly sensitive nanoscale optical metrology platforms. Indeed, the yield of advances in lithography is directly dependent on the availability of rapid, noninvasive metrology that is pertinent to the dimensions of the lithography process at hand. There is thus a very large interest in performing optical metrology with subnanometer resolution but using visible optical wavelengths.

The field of nanophotonics excels at controlling light–matter interactions, scattering, and diffraction from nanoscale structures.^12^ Thereby, it holds an important role in refining the capabilities of optical metrology. Optical metrology poses the interesting design question of which scattering structures and scattering mechanisms provide the most sensitivity to a metrological parameter of interest and which readout scheme is optimal for a given scattering structure. At the same time, the field of nanophotonics develops new microscopy techniques such as superresolution localization microscopy,^3,4,13−15^ Fourier microscopy,^16−18^ and microscopy with structured light^19,20^ that may be leveraged for metrology. As examples of works on the interface of metrology and nanophotonics, indeed, single nanoparticles and oligomers of nanoparticles have proven to be excellent displacement sensors by exploiting the nanoscale interactions between a structured optical field with underlying resonant modes in the nanoparticles,^21−23^ exemplifying the potential of plasmonic and Mie resonances. Metasurfaces are 2-dimensional dense arrays of individually tailored subwavelength designer building blocks,^24^ and can be used to alter fundamental properties of an impinging electromagnetic wave at will.^25,26^ This flexible engineering at the nanoscale has already been leveraged in several optical metrology scenarios. For instance, if the relevant property to be estimated is an overall displacement of a specimen relative to a reference platform, Yuan et al.^27^ reported a metasurface that generates electromagnetic fields with strong phase gradients that may serve as deeply subwavelength markers on a ruler, containing subnanometer displacement resolving power. This approach of strong phase gradients and singularities can also be used with random speckle patterns that can be generated in multimode fiber probes.^28^ Zang et al.^29^ reported a nanometric displacement platform in which absolute metasurface displacements are transduced into polarization rotations, which are subsequently converted into measurable intensity differentials.

In this work, we propose Pancharatnam-Berry (PB) metasurfaces for so-called “interlaced metrology”. Interlaced metrology is a branch of semiconductor optical metrology that retrieves potential misalignments between structures and devices that are written in a multistep lithography process but in the same device layer. For such a metrology use case, typically devoted scattering targets are printed as telltale sensors onto every write field of a device wafer. These targets are designed to be used in combination with diffraction-based readout techniques^30,31^ to sense potential intralayer displacements between nanostructures. A typical approach is that the scattering targets are conventional diffraction gratings consisting of lines or grooves where a first set of lines is written in the first exposure, while in the second exposure, a second set of lines is defined to appear in between the first set. The task of interlaced metrology is to resolve subnanometer displacements of the second set of lines relative to their nominally ideal position as referenced to the first set of lines. This capability often depends on a very precise dictionary of computed or measured scattering efficiencies versus displacement. Such structures do not exploit the full potential of the nanophotonic toolbox. In this work, we present a novel nanometer interlaced metrology sensing platform based on diffractive metasurfaces consisting of anisotropic birefringent meta-atoms. The main idea is to combine two mechanisms to program geometrical sensitivity onto diffraction efficiencies: these are the Pancharatnam-Berry phase^32−35^ on the one hand, and the so-called detour phase^36^ on the other hand. The detour phase effect is well known in grating physics^36^ and computer hologram design^36−38^ and was introduced to the metasurface community by Khorasaninejad et al.^39^ It is an effect wherein meta-atom displacements relative to an underlying periodic lattice induce displacement-dependent phase shifts in scattering. The use of birefringent meta-atoms allows use of the Pancharatnam-Berry phase to magnify the visibility of the displacements. Upon excitation with circularly polarized light, the Pancharatnam-Berry phase principle efficiently transduces interstructural displacement information into measurable polarization splittings in the far-field diffraction channels. In contrast with existing displacement sensing platforms, our concept is fully generic and does not rely on meticulous meta-atom designs to obtain the desired signal. Practically any material of choice could be used, with the only requirement being a structural form birefringence within the meta-atoms. No complex experimental setups or structured illumination conditions are required; a standard Fourier microscope equipped with polarizing optics is sufficient for a successful measurement. In fact, our method can be directly implemented within existing state-of-the-art commercial diffraction-based metrology systems.^10^ The specific polarization signature has the additional advantage that the optical signal can be accurately nulled by polarization analysis and can therefore provide a background-free response. We propose a simple theoretical model elucidating the sensing mechanism and demonstrate all of the qualitative expectations of this model experimentally by performing polarimetric measurements on anisotropic diffractive metasurfaces fabricated from all-dielectric silicon meta-atoms. We show that the polarimetric response encodes deep subwavelength displacements, and we analyze efficient sensing scenarios capable of resolving nanometer-scale displacements, in line with modern-day semiconductor metrology standards. We finally discuss strategies to optimize metasurfaces for maximal sensing performance in the sense of presenting the highest Fisher information assuming a shot noise-limited readout. We show proof-of-concept experiments in which the metrology platform is extended toward two-dimensional (2D) interlaced displacement sensing.

## Theoretical Concept

**Figure 1:**
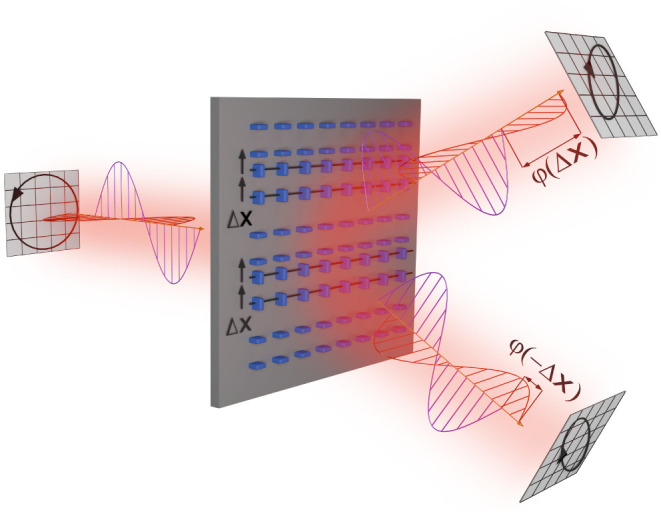
Concept of the metasurface interlaced displacement sensor. A metasurface consisting of alternating lanes of anisotropic meta-atoms is excited by a circularly polarized plane wave. Deeply subwavelength displacements between the lanes of the metasurface lead to an interplay of Pancharatnam-Berry and detour phase shifts in the diffraction channels, which result in a detectable splitting in the ellipticity between the diffraction orders.

**Figure 2:**
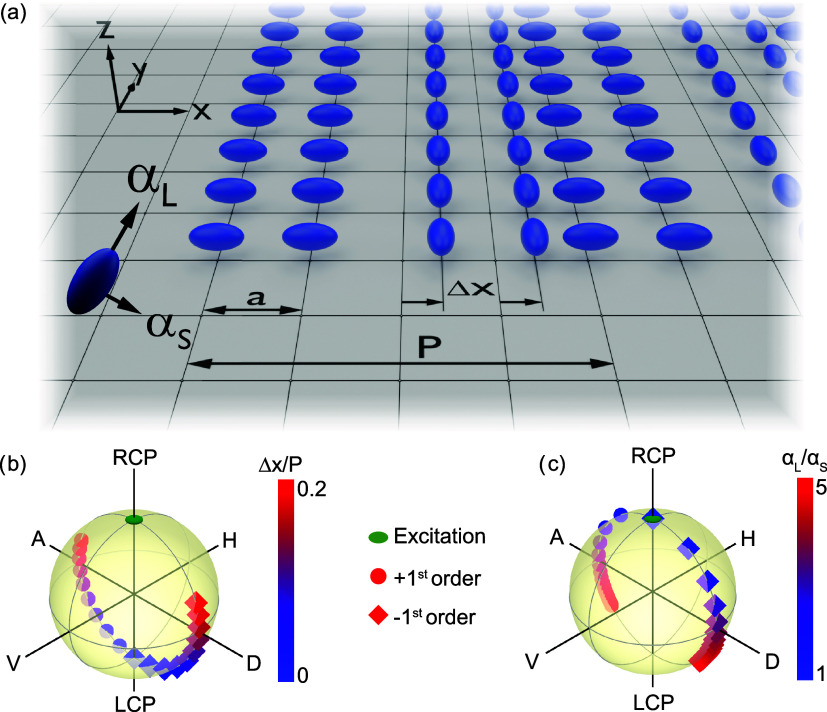
Analytical calculations on anisotropic metagratings show polarization splitting in diffraction orders. (a) Schematic illustration of the metasurface interlaced displacement sensor. (b) Polarization state of the diffraction orders as a function of the normalized displacement  for fixed polarizability ratio . (c) Polarization state of the diffraction orders as a function of the polarizability ratio  for fixed normalized displacement . The green dotted line highlights the polarization state of the excitation beam.

Figure 1 elucidates the main idea of this work: we consider metasurfaces consisting of alternating lanes of birefringent meta-atoms, where from lane to lane the meta-atoms are rotated by degrees of 90°. Upon circularly polarized excitation, the grating diffraction orders must be polarized with an opposite handedness with respect to the excitation if the alternating lanes are exactly equally spaced, a fact that underlies the design philosophy of Pancharatnam-Berry phase metasurfaces. Any displacement Δ*x* of one set of lanes relative to the other set will cause additional symmetry-broken phase shifts φ(±Δ*x*) between the orthogonal polarization components within the diffraction orders as a consequence of the so-called detour phase mechanism, which will in turn lead to a detectable splitting in polarization ellipticity between the two diffraction orders. To bring out these two mechanisms, we describe our diffractive metasurfaces as arrays of polarizable scattering meta-atoms, which are positioned in a lattice with interparticle spacing *a* as depicted in Figure 2a. The total periodicity *P* is equal to *P* = *Na*, depending on the number of participating meta units *N* per unit cell, which is chosen such that it will generate diffraction orders. In this work we limit ourselves to unit cells of 50% duty cycle, meaning that a lane of *N*/2 anisotropic meta-atoms of one orientation is combined with a second lane of *N*/2 meta-atoms that are 90° rotated but otherwise identical. An in-general unknown displacement Δ*x* will be imprinted on the lanes of rotated meta-atoms, which is the quantity we would like to retrieve by measuring the polarization state of the diffraction orders.

The scattering properties of an isolated meta-atom are modeled by an electric dipole polarizability tensor , with α_L_ and α_S_ the polarizabilities of the scatterer along the long and short axes, respectively, accounting for linear birefringence. The polarizability in the out-of-plane dimension is omitted from the formalism, as the normally incident field has no out-of-plane component. It can be shown (see the Supporting Information) that the scattered fields of this anisotropic metagrating in the *m*-th diffraction order in Fourier space can be described by the following expression:
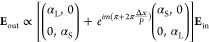
1This expression is derived under two assumptions. First, we assume the first Born approximation, essentially ignoring multiple scattering between meta-atoms. Second, we assume that individual meta-atoms radiate their electric far-field parallel to their dipole moment, which is strictly true only in the paraxial limit of small diffraction angles. Both assumptions could be relaxed by including Ewald lattice sums to deal with meta-atom interactions and by including an orientation-dependent matrix as a prefactor to the right-hand side to account for the high-angle dipole radiation pattern. The scenario of zero displacement Δ*x* = 0 would result in a phase factor of π in the second expression. The condition of nonbirefringent meta-atoms (α_L_ = α_S_) leads to a vanishing of the diffractive signal. This is immediately evident since in this scenario only the lattice with subdiffractive spacing *a* is left. As soon as the meta-atoms are birefringent or as soon as a displacement Δ*x* is introduced, diffraction can occur.

Next, we consider excitation of the metasurface by a circularly polarized plane wave with polarization state described by the Jones vector , with σ = ± *i* for RCP and LCP (right- and lefthanded circular polarization, respectively) illumination, respectively. Supposing we inspect only the ±1 diffraction orders, the diffracted fields can be written as
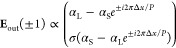
2Notice here how a nonzero displacement Δ*x* introduces a phase slip of  to the diffracted fields, which is opposite in sign for the ± first orders. This phase shift, which is related to the concept of detour phase,^37,38^ thus transduces displacement information in the metasurface plane to polarization information in Fourier space, and is the main carrier of displacement information.

To clarify how the Pancharatnam-Berry phase is leveraged to encode displacements in the polarization state of diffracted orders, we project eq 2 onto a detection of the basis of circular polarization using the Jones calculus. This leads to the circularly copolarized and cross-polarized signals relative to the incident handedness
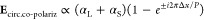
3
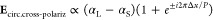
4Notice here that for Δ*x* = 0, only cross-polarized diffraction is present with respect to the excitation field, with a diffraction strength proportional to the linear birefringence. Diffraction in this regime is purely due to the Pancharatnam-Berry (PB) phase,^32,33^ which is a geometric phase that encodes phase shifts purely onto circularly polarized states of light with an opposite handedness with respect to driving fields. For nonzero values of Δ*x*, a copolarized diffraction component is present in the diffracted signal, which can be read out in a polarimetric measurement scheme. A clear indication that this polarimetric scheme can have a sensitivity advantage is that this copolarized displacement-dependent signal occurs on top of a zero-background signal at Δ*x* = 0.

To elucidate the diffractive properties of the anisotropic metasurface that is contained in the preceding analysis, we plot the full polarization state of the two diffracted channels on the Poincaré sphere for two scenarios. First, in Figure 2b, the polarization states of the ±1^st^ orders are plotted versus the normalized displacement parameter  and at a fixed birefringence defined by the polarizability ratio  (set to 3 for this example). For , the polarization states of the two diffraction orders are equal, with a purely circular polarization state with opposite handedness with respect to the excitation, which is due to the aforementioned Pancharatnam-Berry phase. For increasing values of , the polarization states of the diffraction orders split in opposite directions on the Poincaré sphere, which is due to displacement-induced detour phase shifts in eq 2. This combination of Pancharatnam-Berry and detour phase shifts thus allows for a unique encoding of potential deep subwavelength displacements within the plane of the metasurface onto the diffracted polarization states.

To highlight the polarization dependence as a function of the linear birefringence of the meta-atoms, we show the polarization dependence as a function of the polarizability ratio  for a fixed displacement  in Figure 2c. Here, the interplay between Pancharatnam-Berry and detour phase shifts becomes evident. When no birefringence is present in the meta-atoms (), the polarization state of the diffraction orders retains the same handedness state as the excitation field, which is the regime of pure detour phase diffraction, as is also evident from eqs 3 and 4. As the birefringence increases, a cross-polarized contribution is added to the diffracted signal, again resulting in a splitting toward the opposite pole of the Poincaré sphere.

## Experiment

**Figure 3:**
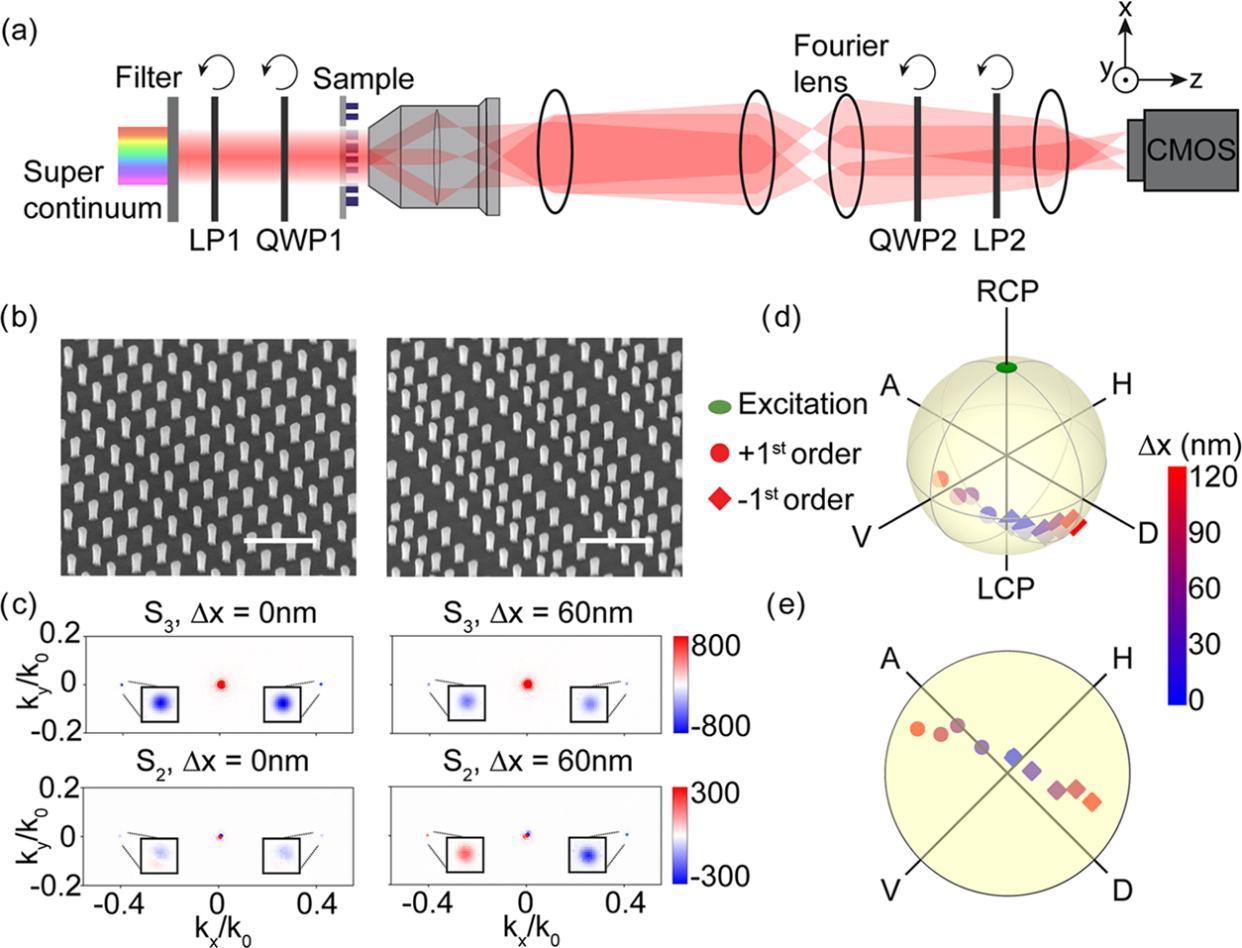
Experimental realization of the PB/detour metasurface interlaced displacement sensor. (a) Fourier space polarimetric microscope for reading out the polarization state of the metasurface. LP: linear polarizer; QWP: quarter wave plate. (b) Tilted view (tilt angle: 45°) scanning electron microscopy images showing undisplaced (Δ*x* = 0 nm, left) and displaced (Δ*x* = 120 nm, right) lanes of a metasurface containing anisotropic meta-atoms. The scale bar corresponds to 1 μm. (c): Third and second Stokes parameters of the diffraction orders of a metasurface with Δ*x* = 0 nm (left) and Δ*x* = 60 nm (right). (d) Full polarization state of the diffraction orders of the metasurface as a function of Δ*x*. The green dot highlights the polarization state of the excitation beam. (e) Two-dimensional projection of the data in (d) onto the lower hemisphere of the Poincaré sphere.

For an experimental realization of the polarimetric displacement sensor, we have fabricated silicon metasurfaces with anisotropic meta-atoms by electron beam lithography (see the Sample Design and Nanofabrication section). The dimensions are chosen such that the meta-atoms act as effective half-wave retarders. This design replicates a meta-atom design by Wang et al.^40^ for PB-phase-based metasurfaces, and the design choice maximizes the PB phase conversion efficiency and thereby fixes the linear birefringence. We note that this is by no means an optimized design for metrology and discuss potential routes for metasurface optimization in a later section. We fabricated several versions of this anisotropic metasurface with different values of the displacement Δ*x*. Scanning electron microscope (SEM) images of typical devices with Δ*x* = 0 and 120 nm are shown in Figure 3b. To analyze the polarization state of the diffraction orders, we utilize Fourier space polarimetry,^16^ with a typical experimental setup shown in Figure 3a. In this technique, the back-focal-plane (BFP) of a microscope objective is imaged onto a CMOS camera, which retrieves the Fourier space of the scattered light from the metasurface and therefore directly images the discrete diffraction channels. A combination of linear polarizers and quarter wave plates in both the input beam and detection path provides control over the polarization state of the input beam, which illuminates the metasurface, and provides the means to interrogate the full polarization state of the scattered signal through Stokes polarimetry. The Stokes parameters *S*_0_, *S*_1_, *S*_2_, and *S*_3_ are determined by acquiring 6 intensity images for different configurations of the collection linear polarizer and quarter wave plate, following the protocol outlined in ref (16). The first Stokes parameter *S*_0_ corresponds to the total intensity of the acquired signal, while the remaining three Stokes parameters report intensity differentials between orthogonal polarization states: *S*_1_ for the horizontal/vertical basis, *S*_2_ for the diagonal/antidiagonal basis, and *S*_3_ for the lefthanded/righthanded circular basis.

In the following experiments, we perform Fourier space polarimetry at a wavelength of 650 nm using metasurfaces with interparticle spacing *a* = 400 nm and 4 meta units per unit cell (*N* = 4). In Figure 3c, raw images of the Stokes parameters *S*_3_ and *S*_2_ of the diffraction channels of unperturbed (Δ*x* = 0 nm, left) and perturbed (Δ*x* = 60 nm, right) metagratings are shown. First, we note that the *S*_3_ parameter of the ± first diffraction orders at zero displacement is fully sign reversed with respect to the direct transmission of the beam through the sample. This observation highlights the PB phase shift due to the anisotropic meta-atoms. Since at zero displacement these orders are fully circularly (cross-) polarized, they carry no preferential linear polarization, leading to a null signal in *S*_2_. In contrast, for the displaced version of the metasurface, the *S*_2_ parameter shows a strong diffraction signature. This splitting in *S*_2_ is precisely due to the additional detour phase shift provoked by the interstructural displacements in the plane, as predicted by our model, causing marked ellipticity. The nonzero *S*_2_ parameter not only directly visualizes that there is a nonzero displacement Δ*x* but also through its sign directly reveals the direction of the shift.

To summarize the complete polarization dependence as a function of displacement, we show the measured polarization states of the diffraction orders for metasurfaces with different values of displacement on the Poincaré sphere in Figure 3d. Here, we recover the same trend of polarization splitting as predicted by our model, as shown in Figure 2b. We also note here that the trajectory of the measured splittings on the Poincaré sphere is not exactly in the *S*_1_ = 0 plane, and after a 2D projection of the data points onto the lower hemisphere of the Poincaré sphere in Figure 3e an additional minor splitting is clearly present in the *S*_1_ parameter as well. This can be explained by the fact that slight deviations from the ideal half-wave retarder condition in the Si meta-atoms cause an additional phase lag between the scattering polarizabilities α_L_ and α_S_, leading to nonsymmetric splitting effects on the Poincaré sphere (see the Supporting Information).

## Metrology and Responsivity

While analyzing excursions of the diffracted orders on the Poincaré sphere highlights the essential physics of encoding displacements through detour and PB phase, it arguably does not constitute a practical measurement scheme for metrology. In this section, we analyze polarimetric measurement schemes that are more applicable in an interlaced metrology scenario. Current day requirements for semiconductor interlaced metrology involve displacement sensitivity down to the nanometer scale, while maintaining a high inspection throughput, corresponding to measurement times of approximately 100 ms per target.^10^ Reading out the full polarization state by sequential image recordings using mechanically rotating polarizing optics is, in that context, not very practical. A fast readout scheme using specific combinations of QWP2 and LP2, which optimally converts the polarization characteristics into intensity differentials, is, therefore, desirable.

**Figure 4:**
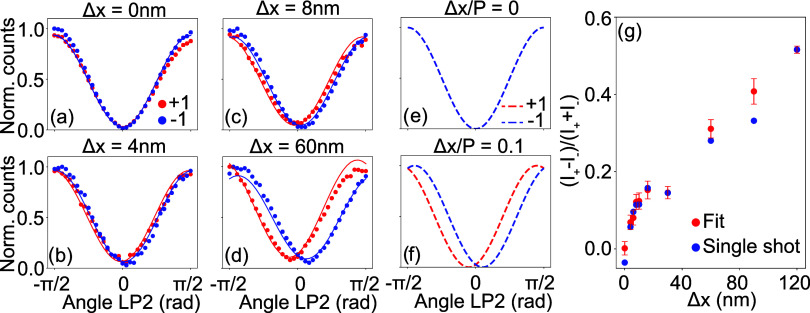
Application of the anisotropic metasurface in an interlaced metrology scenario. Measurements and corresponding fits of shifted sin^2^(Θ) functions of the intensities of the ±1st diffraction orders for fixed angle of QWP2 (45°), while rotating LP2 by angle Θ for a metasurface imprinted by (a): Δ*x* = 0 nm, (b): Δ*x* = 4 nm, (c): Δ*x* = 8 nm, and (d): Δ*x* = 60 nm. (e, f) Analytical calculations of metasurfaces containing normalized displacement parameters  and , respectively. (g) Responsivity of the normalized intensity differential estimator versus metasurface displacement Δ*x* extracted from a single measurement (blue) and the fit results (red).

To investigate optimal measurement protocols, we measure diffraction intensities of the metagratings by keeping the incident polarization circular and fixed, while on the analysis side fixing the angle of the quarter wave plate QWP2 to 45° relative to the *y*-axis in Figure 3a, while rotating the angle of the linear polarizer LP2 from 0 to 180°. The results of this measurement are shown in Figure 4 for metasurfaces with encoded displacements of Δ*x* = 0 nm in panel (A), Δ*x* = 4 nm in panel (B), Δ*x* = 8 nm in panel (C), and Δ*x* = 60 nm in panel (D). First, we note that the diffracted intensities follow a sinusoidal behavior with respect to the angle Θ of LP2, where in the case of Δ*x* = 0 nm maximum signal is measured when LP2 is fixed at 0 and 180°, while no signal is measured at an angle of 90°. These regimes correspond to circular polarization filtering of, respectively, the orthogonal and parallel handedness states with respect to the excitation, highlighting the well-known PB phase effect in grating diffraction. Furthermore, a clear symmetrical shift between the sinusoids of the ± first diffraction orders around Θ = 90° is observed which increases as a function of Δ*x*. Indeed, fitting our experimental data with a pair of sin^2^(Θ) functions that are symmetrically shifted relative to the 90° point results in an excellent match. It is easy to verify that the behavior of these experimental curves is consistent with our simple model by applying Jones calculus to the diffracted fields predicted by eq 2, as shown for displacement parameters  in panel (E) and  in panel (F). These results show that displacements even down to the few nanometer scale can be resolved and that the displacement is essentially encoded in the fitted shift of the null signal away from Θ = 90°.

For metrology, a suitable estimator needs to be defined that relates displacements within the plane of the metasurface into differentials of measurable observables. To keep up with the speed requirements of modern-day interlaced metrology, we propose an estimation scheme relying on acquisition at a single polarizer setting instead of fitting the sinusoidal dependencies evident in Figure 4 in full. The idea is to measure at the fixed angle of LP2 at which the maximum intensity difference between the plus and minus first diffraction orders occurs, which by construction is at Θ = ± 45° (independent of displacement). For the data set at hand, the blue data points in Figure 4g report the intensity difference measured for metasurfaces with different lithographically imprinted displacements. More precisely stated, we work with a normalized intensity difference Δ*I*_±_ that divides out absolute intensities and grating efficiencies, defined as
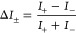
5where *I*_±_ represents the intensity in the positive and negative diffraction orders. For comparison, in the same graph, we indicate Δ*I*_±_ as extracted from the fit results by red data points, where the errorbars are deduced by propagating the errors from individual fit parameters, and achieve a good overlap with the normalized intensities as extracted from the single measurement. Selecting the range Δ*x* ≤ 20 nm, we deduce a responsivity of  percent intensity change per nanometer displacement.

This estimation scheme has, apart from being high speed, several advantages for semiconductor metrology. First, the measurement is inherently self-referencing to potential fluctuations in laser power or differences in scattering strengths of different metasurface configurations, which might cause overall fluctuations in absolute diffracted counts, as is also evident from our measurements. Second, metrology sensors are usually closely surrounded by other structures on the device wafer which might corrupt the sensing signal by scattering crosstalk.^41^ Because the relevant signal here relies on specific polarization filtering, we expect the unwanted crosstalk to be effectively filtered out.

**Figure 5:**
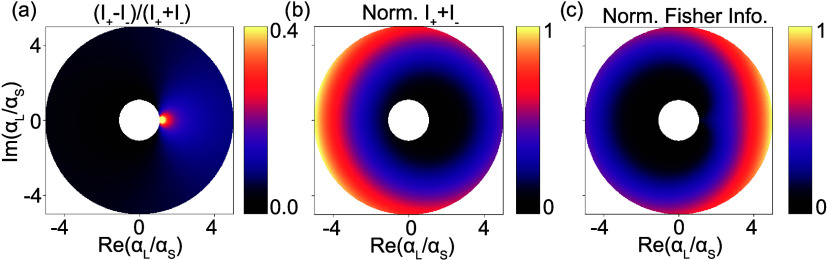
Meta-atom optimization and noise considerations. As a function of polarizability ratio , in the complex plane, we show: (a) normalized responsivity parameter , (b) diffraction efficiency *I*_+_ + *I*_–_, and (c) Fisher information on a measurement while using the readout strategy discussed in the Metrology and Responsivity section.

All experimental results shown thus far have been conducted with fixed meta-atom polarizabilities satisfying half-wave plate conditions,^40^ which are optimal for Pancharatnam-Berry metasurface diffraction efficiencies, but which are not necessarily optimal for sensing performance. To investigate how the meta-atoms can be optimized, we turn again to our analytical model and calculate diffraction intensity contrast Δ*I*_±_ for fixed angles of QWP2 and LP2, as defined for the single shot metrology scenario in Figure 4f, and for a fixed normalized displacement parameter . For different values of the meta-atom polarizability ratio , we calculate values of the normalized differential intensity estimator Δ*I*_±_ and plot the results in the complex polarizability plane in Figure 5a. From this figure alone, it might appear that the optimal polarizability ratio approaches unity (no birefringence), where Δ*I*_±_ results in a maximum responsivity. This, however, overlooks the fact that in precisely this regime, the PB diffraction efficiency is minimal, which results in a low number of detected photons and thus a low signal-to-noise ratio in an experimental setting. This is evident from Figure 5b, where we plot the total diffraction intensities of the ±1st diffraction orders. It is thus evident that in light of noise and photon budget, there is a trade-off between optimizing absolute diffraction efficiency on one hand, and the relative contrast Δ*I* on the other hand.

We use a concept from information theory called “Fisher information”^42^ to analyze this trade-off in light of noise. In brief, the Fisher information quantifies the amount of information a measured data set contains with respect to a relevant parameter and puts a bound on the maximum precision with which this parameter can be estimated given certain noise statistics assumed for the observable at hand. This concept has already been successfully applied in several metrology scenarios, such as optimizing a metagrating for transverse position metrology^43^ and finding optimal illumination states in coherent scattering experiments.^44^ Assuming for simplicity that photon shot noise is the limiting noise factor, we can derive (see the Supporting Information) an explicit expression for the Fisher information in our specific measurement protocol according to
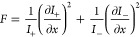
6and plot its values in the complex plane of polarizability ratios in Figure 5c. Comparing this figure to the regime in which our metasurface operates, which is the negative direction on the real axis of the polarizability ratio (due to the half-wave plate condition), it is seen that the sensing performance of the metasurface can still be optimized substantially. The results presented in this work thus only report a very conservative bound on the displacement resolving power of our metasurface, and we expect that subnanometer precision lies within reach.

## 2D Metagratings

**Figure 6:**
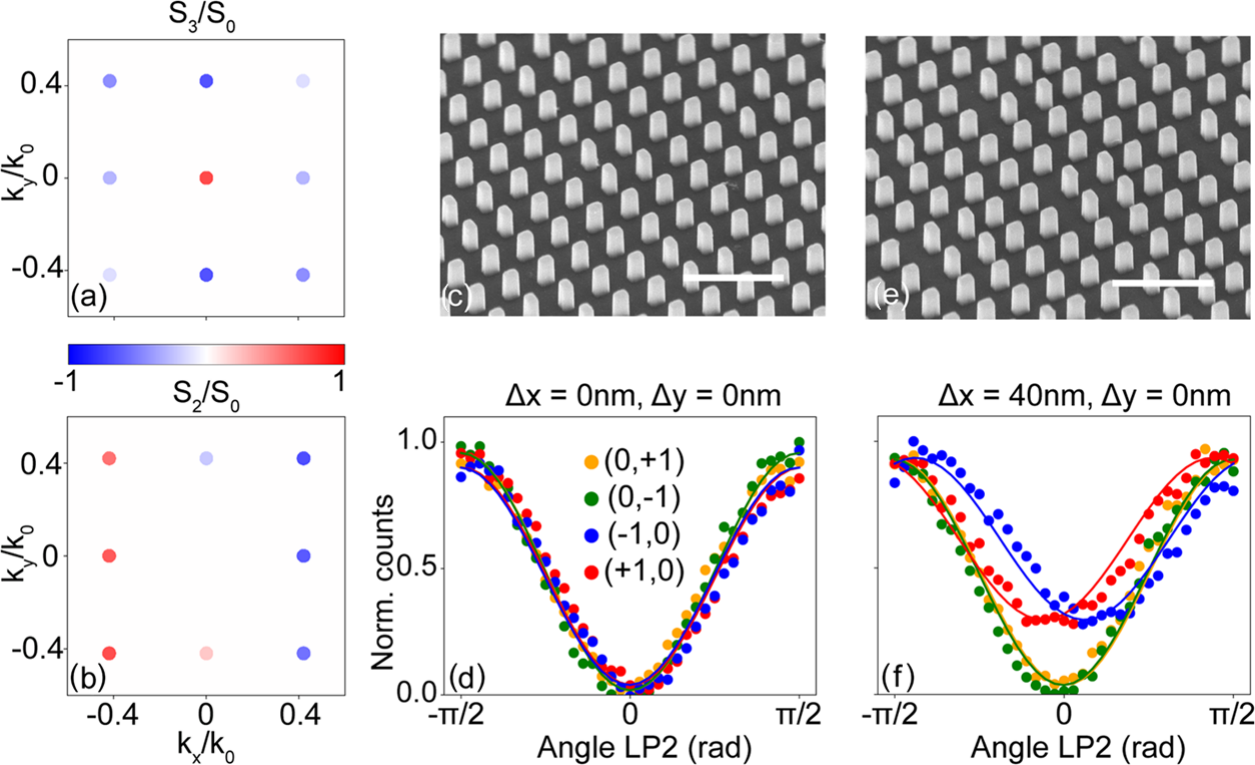
Toward 2D interlaced metrology in anisotropic metasurfaces. (a, b) Calculated normalized Stokes parameters *S*_3_/*S*_0_ and *S*_2_/*S*_0_, respectively, of a 2D anisotropic metasurface ( with  and ). (c, e) Tilted view (tilt angle: 45°) SEM image of an unperturbed (Δ*x* = 0 nm, Δ*y* = 0 nm) and perturbed (Δ*x* = 80 nm, Δ*y* = 0 nm) 2D displacement sensing metasurface, respectively. The scale bar corresponds to 1 μm. (d, f) Measurements on unperturbed (Δ*x* = 0 nm, Δ*y* = 0 nm) and perturbed (Δ*x* = 40 nm, Δ*y* = 0 nm) version of 2D anisotropic metasurfaces, respectively, with corresponding fits of shifted sin^2^(Θ) functions of the intensities of the four diffraction orders for the fixed angle of QWP2 (45°), while rotating LP2 by angle Θ.

Finally, we show proof-of-concept experiments in which the metasurface displacement sensing platform is extended toward interlaced metrology along two directions. For this, we fabricated metagratings with diffractive periodicities in both *x* and *y* directions as shown in the SEM image in Figure 6c,e for an unperturbed (Δ*x* = 0 nm, Δ*y* = 0 nm) and perturbed (Δ*x* = 80 nm, Δ*y* = 0 nm), respectively, which will now generate diffraction channels along both *k*_*x*_ and *k*_*y*_ directions. In the same way, as discussed for the one-dimensional metasurface, arbitrary two-dimensional displacements can be imprinted onto the set of rotated meta-atoms in the unit cell, which will subsequently lead to detour phase shifts in the diffraction channels along the *k*_*x*_ and *k*_*y*_ directions for displacements in *x* and *y*, respectively. Figure 6a,b shows calculated values of normalized *S*_3_ and *S*_2_ Stokes parameters of a typical Fourier image of such a metasurface containing 2D displacements (with , ), where again the PB-phase shift and polarization splitting are evident in *S*_3_ and *S*_2_, respectively. Because the detour phase shifts, and thereby also the strengths of the polarization splitting, imprinted on the diffraction channels in the *k*_*x*_ (*k*_*y*_) viewing directions are specifically linked to the 1-dimensional displacement components *x* (*y*) within the metasurface plane, complete 2D displacement information can be retrieved from a single polarization resolved measurement by analyzing the set of (*k̂*_*x*_, *k̂*_*y*_) = [(−1, 0), (1, 0), (0, −1), (0, 1)] diffraction orders.

To show this directional link between metasurface displacements and polarization splitting in the relevant viewing direction in Fourier space, we perform polarimetric measurements on the four diffraction orders of an unperturbed (Δ*x* = 0 nm, Figure 6d) and perturbed (Δ*x* = 40 nm, Figure 6f) version of a two-dimensional anisotropic metagrating, using the same measurement protocol as was used in Figure 4. The results again show identical intensity dependencies for the unperturbed metagrating, whereas for the perturbed metagrating only the set of ((−1, 0), (1, 0)) orders along the *k*_*x*_ direction show the characteristic shift in intensity curves commensurate with the fact that for this particular sample the displacement vector is nonzero only in the *x*-direction. This data also shows an overall intensity offset, which we attribute to deviations in the meta-atom dimensions from the half-wave plate condition (see the Supporting Information). We further note that linear combinations of the displacements Δ*x* and Δ*y* are transduced not only via the lowest diffraction orders but also into the diagonal diffraction orders (*k̂*_*x*_, *k̂*_*y*_) = [(−1, −1), (1, −1), (1, −1), (1, 1)], which might be analyzed in addition to enhance the signal-to-noise ratio.

## Conclusions

In conclusion, we proposed a sensing platform that encodes deeply subwavelength displacements within a layer of an anisotropic diffractive metasurface onto the polarization signatures of the diffraction orders. We argued that the sensing mechanism relies on an interplay between Pancharatnam-Berry and detour phase shifts and demonstrated that displacements down to a few nanometers can be effectively resolved. A key advantage of our platform is the background-free nature of the signal. Using a specific polarimetric readout strategy, the signal provides an inherently self-referenced estimator for potentially unknown displacements, which renders the knowledge of experimental parameters such as total laser power, integration time, and diffraction efficiencies irrelevant. For an accurate estimation of unknown displacements, however, the polarizability contrasts of the meta-atoms need to be known, which can be inferred from a calibration measurement using a metasurface with a priori known displacement. This highlights an additional advantage of the sensing platform: at known displacements, these structures can also report on the polarization anisotropy and on whether nominally identical but simply rotated polarization anisotropies are indeed identical up to rotation. As such, the presented structures could also be used as optical sensors of shape parameters, e.g., as a reporter for anisotropies and astigmatism in lithography. We expect that the responsivity of the metasurface can be optimized substantially by designing anisotropic meta-atoms with giant polarizability ratios, which could be achieved by for instance exploiting the physics of bound states in the continuum^45^ or inverse design strategies,^46^ and believe that subnanometer resolving power lies within reach. We note that both the detour phase and the PB mechanism are fully generic and require designs that show only structural birefringence. Furthermore, the meta-atoms themselves do not require a minimum size or refractive index. As such, this approach to metrology can work also on arrays of deeply subwavelength structures within each lane, as long as they present optical form birefringence in an effective medium limit. This is of relevance in semiconductor manufacturing, where it is desirable to have metrology target structures that are similar in size to devices at the relevant manufacturing node. Furthermore, we highlight the possibility of extending the method toward aperiodic metrology targets in the form of Pancharatnam-Berry holographic phase masks, which could provide an information advantage in the far-field polarization signal with respect to the sparse discrete diffraction channels as analyzed in this work. Finally, the concepts of Pancharatnam-Berry phase and detour phase are applicable not only to metrologies within a single layer but also to overlay metrologies that aim to resolve lateral displacements between vertically offset layers. Overall, our work provides a metasurface-based platform for retrieving structural misalignments within a device, a highly relevant problem in today’s semiconductor manufacturing processes.

## Methods

### Experimental Setup

A supercontinuum laser (Leukos Electro-VIS) is monochromated (1 nm bandwidth) by an Acousto-Optic Tunable Filter (AOTF) and coupled into a single-mode optical fiber. The output of the fiber (power 120 μW) is subsequently collimated by a lens (Thorlabs, AC-254-035-A) and guided through the first linear polarizer (Thorlabs, LPVICS100-MP2) and quarter wave plate (Thorlabs, AQWP05M-600) to obtain circularly polarized illumination. After passing through the sample, an objective (NIKON, CFI Achro) captures the diffracted signal and is guided through a telescope consisting of two lenses (Thorlabs, AC-254-050-B). The polarization state of the diffracted signal is then analyzed by the second quarter wave plate (Thorlabs, AQWP05M-600) and linear polarizer (Thorlabs, LPVIS100-MP2). The Fourier plane of the metasurface is finally imaged by a last pair of lenses (Thorlabs, AC-254-200-B) onto a CMOS camera (PCO Panda 4.2). All polarization optics were mounted in motorized (Thorlabs, KDC101) rotation mounts (Thorlabs, PRM1/MZ8). For the Stokes polarimetry measurements in Figure 3, the wavelength was set to 630 nm, and 20 frames were averaged with an integration time of 150 μs. In the sensitivity measurements in Figure 4, the wavelength was set to 650 nm, and 20 frames were averaged with an integration time of 20 μs.

### Sample Design and Nanofabrication

We fabricated Si metasurfaces by using electron beam lithography using the following procedure. A fused quartz substrate (Menzel-Gläser) with a thickness of 0.5 mm was sonicated in H_2_O for 10 min and subsequently cleaned in a solution of NH_4_OH/H_2_H_2_/H_2_O = 1/1/5 (base piranha) at 75 °C for 15 min and, after rinsing with H_2_O, blown dry with N_2_. 320 nm of amorphous silicon (a-Si) was deposited onto the substrate using physical vapor deposition (Polyteknik Flextura M506 S) at RF power 260 W with the corresponding deposition rate of 0.12 nm/s. After an oxygen plasma descum process of 2 min, a 160 nm layer of hydrogen silsequioxane (HSQ) was spin-coated (Süss Microtec Delta 80 spin-coater) for 60 s with a speed of 1000 rpm and acceleration of 1000 rpm/s and baked for 2 min at 180 °C. A conductive layer (Espacer 300Z) was spin-coated for 60 s with a speed of 2000 rpm and acceleration of 1000 rpm/s. 50 nm gold nanoparticles were drop-cast in the corners of the substrate for aligning the electron beam (Raith). Exposure of the sample was done with 50 kV acceleration voltage and an e-beam dose of 2200 μC/cm^2^. After exposure, the substrate was developed for 60 s in a solution of tetramethylammonium hydroxide (TMAH) at 60 °C and rinsed with H_2_O for 15 s. Finally, the substrate was etched (Oxford Plasmalab 80+) using a mixture of 10 sccm sulfur hexafluoride (SF_6_), 15 sscm trifluoromethane (CHF_3_), and 3 sccm O_2_. The final anisotropic meta-atoms in the metasurface have a length of 145 nm, a width of 105 nm, and a height of 320 nm.

## Supporting Information Available

Derivation of the diffracted fields of an anisotropic metasurface; phase lags in the polarizability tensor; and derivation of the Fisher information in the experiment (PDF)
