# Supplementary material for: Nanometer Interlaced Displacement Metrology Using Diffractive Pancharatnam-Berry and Detour Phase Metasurfaces
Source: ACS Photonics. 2024 Nov 30;11(12):5229–38. doi: 10.1021/acsphotonics.4c01451 (PMC11660269; doi:10.1021/acsphotonics.4c01451)
Supplement: Supplementary file 1 — ph4c01451_si_001.pdf [file ph4c01451_si_001.pdf]

Supplementary information: Nanometer interlaced displacement metrology using diffractive Pancharatnam-Berry and detour phase metasurfaces

Nick Feldman,[†,‡] Kian M. M. Goeloe,[†] Arie J. den Boef,[‡,¶,§] Lyubov V. Amitonova,[‡,¶] and A. Femius Koenderink*,[†]

†*Department of Information in Matter and Center for Nanophotonics, AMOLF, Science Park 104, 1098 XG, Amsterdam, Netherlands*

‡*Advanced Research Center for Nanolithography (ARCNL), Science Park 106, 1098 XG Amsterdam, The Netherlands*

¶*Department of Physics and Astronomy, and LaserLaB, Vrije Universiteit, De Boelelaan 1081, 1081 HV Amsterdam, The Netherlands*

§*ASML Netherlands B.V., De Run 6501, 5504 DR, Veldhoven, The Netherlands*

E-mail: f.koenderink@amolf.nl

Diffracted fields of an anisotropic metagrating

For didactic purposes, we first consider the scattering behaviour of a metagrating with identical meta-atoms and a duty cycle of $P/2$, where the long axis of the meta-atom is oriented along the principal axis of the grating. The fields scattered by such a grating upon excitation by some incident field $\mathbf{E}_{\text{in}}$ are described by a series of sources:

$$\mathbf{E}_{\text{out}}(\mathbf{r}) = \left(\left(\left(\sum_{m \text{ integer}} \delta(r - ma) \right) \cdot \text{rect(r;-P/4,P/4)} \right) \star \sum_{m \text{ integer}} \delta(r - mP) \right) \begin{pmatrix} \alpha_L, 0 \\ 0, \alpha_S \end{pmatrix} \mathbf{E}_{\text{in}}(\mathbf{r}), \tag{1}$$

where we ignore multiple scattering between individual meta-atoms and thereby assume metasurface operation in the 1st Born approximation. This metagrating will generate diffraction channels in Fourier space, which can be analyzed by Fourier transforming equation 1:

$$\mathbf{E}_{\text{out}}(\mathbf{k}_{||}) = \mathcal{F}[\text{geometry}] \left[\mathbf{M}(\mathbf{k}_{||}) \begin{pmatrix} \alpha_L, 0 \\ 0, \alpha_S \end{pmatrix} \mathbf{E}_{\text{in}} \right]. \tag{2}$$

This expression describes light scattering in Fourier space as the product of the Fourier-transform of the geometry $\mathcal{F}[\text{geometry}]$ with the radiation pattern of the isolated meta-atoms $\mathbf{M}(\mathbf{k}_{||})$. For simplicity, we will assume that the meta-atoms scatter light uniformly in all directions by equating $\mathbf{M}(\mathbf{k}_{||})$ to the identity matrix $\mathbf{I}$. The Fourier transform of the geometry can be evaluated by using the convolution theorem:

$$\mathcal{F}[\text{geometry}] = \left(\mathcal{F}[\sum_{m \text{ integer}} \delta(r - ma)] \star \mathcal{F}[\text{rect(r;-P/4,P/4)}] \right) \cdot \mathcal{F}[\sum_{m \text{ integer}} \delta(r - mP)]. \tag{3}$$

This expression consists of three main parts: the first part describes the Fourier transform of the dense sub-diffractive lattice, which will again result in a discrete lattice in Fourier space. The only k-vector component that is allowed to propagate, however, is the $k_{//} =$

0 component, by virtue of the sub-diffractive spacing a. The second part is the Fourier transform of a rectangle function, which is a sinc function. These two expressions are finally multiplied by the Fourier transform of the supercell lattice, which will generate the relevant diffraction channels. Summarizing these operations, we obtain:

$$\mathbf{E}_{\text{out}}(\mathbf{k}_{||}) \propto \sum_{m \text{ integer}} \text{sinc}\left(\frac{k_{||}P}{4}\right)\delta\left(\mathbf{k}_{||} - m\frac{2\pi}{P}\right)\left[\begin{pmatrix}\alpha_L, 0\\ 0, \alpha_S\end{pmatrix}\mathbf{E}_{\text{in}}\right]. \tag{4}$$

The metagrating studied in the main text is the sum of two gratings with identical periodicity and duty cycle, but where in the second grating the scattering objects are differently oriented (90 degrees rotated) with respect to the objects in the first grating, which can be described by rotating the polarizability tensor to $\begin{pmatrix}\alpha_S, 0\\ 0, \alpha_L\end{pmatrix}$. Moreover, the second grating will experience a shift with respect to the first grating, where the shift is in general equal to $P/2 + \Delta x$. Due to the Fourier shift theorem, the diffracted fields in Fourier space will experience an additional phase shift due to this displacement in real space, such that the total diffracted fields of the composite metagrating now read:

$$\mathbf{E}_{\text{out}}(\mathbf{k}_{||}) \propto \sum_{m \text{ integer}} \text{sinc}(k_{||}P/4)\delta(\mathbf{k}_{||} - m\frac{2\pi}{P})\left[\begin{pmatrix}\alpha_L, 0\\ 0, \alpha_S\end{pmatrix} + e^{ik_{||}(P/2+\Delta x)}\begin{pmatrix}\alpha_S, 0\\ 0, \alpha_L\end{pmatrix}\right]\mathbf{E}_{\text{in}}. \tag{5}$$

Notice how in the scenario of $\Delta x = 0$, the extra phase shift in the diffracted fields of the second grating would be equal to -1, due to the diffraction condition $k_{||}P = 2\pi m$ in the $m'th$ diffraction channel. If the building blocks would be identical ($\alpha_L = \alpha_S$, then the diffraction channels would vanish, due to perfect destructive interference of the two interspersed gratings. This is logical, since in that case there only is a sub-diffractive lattice left.

To arrive at the expression from the main text, we substitute the grating condition into

equation 5 to obtain:

$$\mathbf{E}_{\text{out}} \propto \left[\begin{pmatrix} \alpha_L, 0 \\ 0, \alpha_S \end{pmatrix} + e^{im(\pi + \frac{2\pi\Delta x}{P})} \begin{pmatrix} \alpha_S, 0 \\ 0, \alpha_L \end{pmatrix} \right] \mathbf{E}_{\text{in}}. \tag{6}$$

Extension to 2D metasurfaces

Considering the two dimensional metagrating as discussed in figure 6 from the main text, with 3 unrotated meta-atoms and 1 rotated meta-atom in the unitcell, the diffracted fields can be extended to two dimensions:

$$\mathbf{E}_{\text{out}}(\mathbf{k}_x, \mathbf{k}_y) \propto \sum_m \sum_n \delta\left(\mathbf{k}_x - m\frac{2\pi}{P}\right)\delta\left(\mathbf{k}_y - n\frac{2\pi}{P}\right) \tag{7}$$

$$\left[\left(1 + e^{i\frac{k_x P}{2}} + e^{\frac{ik_y P}{2}}\right)\begin{pmatrix}\alpha_L, 0\\ 0, \alpha_S\end{pmatrix} + e^{ik_x\left(\frac{P}{2}+\Delta x\right)} e^{ik_y\left(\frac{P}{2}+\Delta y\right)}\begin{pmatrix}\alpha_S, 0\\ 0, \alpha_L\end{pmatrix}\right]\mathbf{E}_{\text{in}}.$$

Phase lags in the polarizability tensor

In this section, we show how deviations from the ideal half wave plate condition can lead to non-symmetrical polarization splitting effects on the Poincaré sphere and intensity offsets in metrological experiments such as we report in figures 4 and 6 in the main manuscript. For this, we introduce extra phase lags between the complex polarizabilities α_L and α_S and calculate the polarization states of the diffraction orders and corresponding sensitivity curves using our analytical model. In this particular example, we set α_S to 1 and α_L to $2.25e^{1.25\pi i}$. Figure S1a shows the corresponding polarization state of the diffraction channels on the Poincaré sphere, and figure S1b shows the corresponding 2D projection onto the lower hemisphere, showing marked deviations from the symmetric splitting in S_2 such as we measured in experiments. For the same polarizability settings, we furthermore plot the analytical sensitivity curves using the same procedure as was done in figures 4 and 6 for an unperturbed ($\frac{\Delta x}{P} = 0$) and perturbed ($\frac{\Delta x}{P} = 0.25$) metasurface. Here we note an intensity offset in the shifted sin^2 functions, such as we have also observed in figure 6f and 4d in the main text.

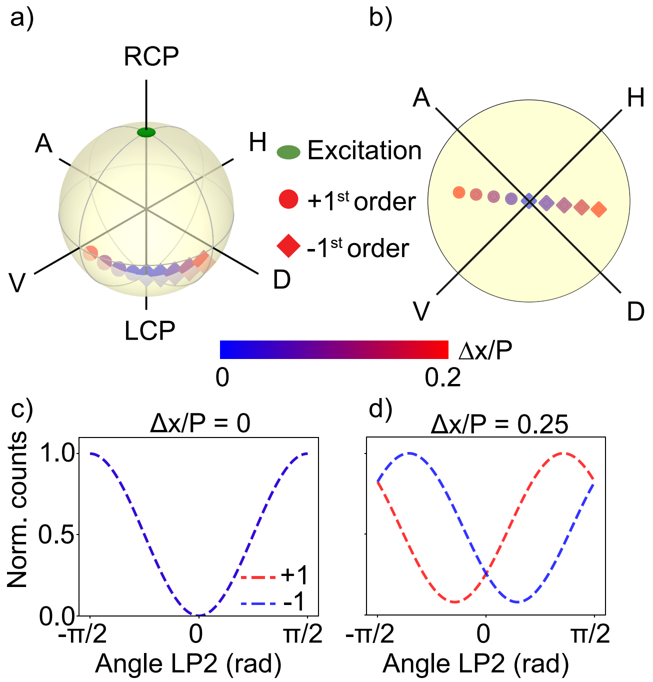

Figure S1: **Influence of phase lags in the complex polarizability tensor.** Panel (a): Polarization state of the diffraction orders on the Poincare sphere for $\alpha_S = 1$ and $\alpha_L = 2.25e^{1.25\pi i}$. Panel (b): 2D projection of the data in panel (a) onto the lower hemisphere of the Poincare sphere. Panels (c) and (d) show corresponding sensitivity curves for $\frac{\Delta x}{P} = 0$ and $\frac{\Delta x}{P} = 0.25$ respectively.

Fisher information of a single pixel measurement

To derive the information content in one diffraction channel of the anisotropic metasurface, we derive the Fisher information of a single pixel measurement. The Fisher information is defined according to:[1]

$$F = E\left[\left(\frac{\partial \ln p(X;\hat{\Theta})}{\partial \hat{\Theta}}\right)^2\right], \tag{8}$$

where $E[]$ is the expectation operator, $p(X;\hat{\Theta})$ is a probability density function, describing the probability of measuring a dataset X parametrized by an in principle un-known parameter $\hat{\Theta}$. In our work, this parameter $\hat{\Theta}$ corresponds to potential displacements Δx in the plane of the metasurface, which we would like to estimate based on our polarimetric measurement. The Fisher information then quantifies a lower bound on the variance with which

this unknown parameter can be estimated, according to the Cramér-Rao lower bound:

$$V(\hat{\Theta}) \geq \frac{1}{F}. \tag{9}$$

We assume that shot-noise is the limiting noise contribution in our measurements. In that case, $p(X;\hat{\Theta})$ follows Poisson statistics:

$$p(X = k; \hat{\Theta}) = \frac{I^k e^{-I}}{k!}, \tag{10}$$

which describes the probability of measuring k photons onto a single pixel detector and $I(\hat{\Theta})$ the mean number of photons. Substituting this expression for the proability density into equation 8 and working out the terms results in:

$$F = \left(\frac{\partial I}{\partial \hat{\Theta}}\right)^2 \left(\frac{1}{I^2} E\left[k^2\right] - \frac{2}{I} E\left[k\right] + E\left[1\right]\right). \tag{11}$$

Here, we recognize the moments of the expectation operator:

$$\begin{aligned} E\left[k^2\right] &= I^2 + I \\ E\left[k\right] &= I \\ E\left[1\right] &= 1 \end{aligned} \tag{12}$$

and obtain the expression for the Fisher information content from a shot-noise limited single pixel bucket measurement:

$$F = \frac{1}{I}\left(\frac{\partial I}{\partial \hat{\Theta}}\right)^2. \tag{13}$$

In order to apply this expression to our Fourier space polarimetric readout scheme, we notice that our parameter estimation problem relies on measuring the intensities of 2 discrete

diffraction orders, which can, in principle, be acquired by two single pixel bucket detectors. Because the Fisher information is an additive quantity, the information content in our polarimetric measurement can finally be written as:

$$F = \frac{1}{I_+}\left(\frac{\partial I_+}{\partial x}\right)^2 + \frac{1}{I_-}\left(\frac{\partial I_-}{\partial x}\right)^2, \tag{14}$$

where $I_\pm$ and $\frac{\partial I_\pm}{\partial x}$ are the diffracted intensity and intensity differentials respectively in the $\pm 1^{st}$ diffraction orders

References

(1) Kay, S. M. *Fundamentals of statistical signal processing: estimation theory*; Prentice-Hall, Inc., 1993.
